# Novel hole-pillar spacer design for improved hydrodynamics and biofouling mitigation in membrane filtration

**DOI:** 10.1038/s41598-021-86459-w

**Published:** 2021-03-26

**Authors:** Adnan Qamar, Sarah Kerdi, Syed Muztuza Ali, Ho Kyong Shon, Johannes S. Vrouwenvelder, Noreddine Ghaffour

**Affiliations:** 1 King Abdullah University of Science and Technology (KAUST), Water Desalination and Reuse Center (WDRC), Biological and Environmental Science and Engineering (BESE), Thuwal, 23955-6900 Saudi Arabia; 2 School of Civil and Environmental Engineering, University of Technology, Sydney, Post Box 129, Broadway, NSW 2007 Australia

**Keywords:** Environmental sciences, Energy science and technology, Engineering

## Abstract

Feed spacers are the critical components of any spiral-wound filtration module, dictating the filtration performance. Three spacer designs, namely a non-woven commercial spacer (varying filament cross-section), a symmetric pillar spacer, and a novel hole-pillar spacer (constant filament diameter) were studied using Direct Numerical Simulations (DNS), 3-D printed and subsequently experimentally tested in a lab-scale ultrafiltration set-up with high biofouling potential feed water at various feed pressures. Independent of the applied pressure, the novel hole-pillar spacer showed initially the lowest feed channel pressure drop, the lowest shear stress, and the highest permeate flux compared to the commercial and pillar spacers. Furthermore, less biofilm thickness development on membrane surface was visualized by Optical Coherent Tomography (OCT) imaging for the proposed hole-pillar spacer. At higher feed pressure, a thicker biofilm developed on membrane surface for all spacer designs explaining the stronger decrease in permeate flux at high pressure. The findings systematically demonstrated the role of various spacer designs and applied pressure on the performance of pre-treatment process, while identifying specific shear stress distribution guidelines for engineering a new spacer design in different filtration techniques.

## Introduction

Feed spacer is one of the critical components of the spiral wound membrane modules, which effectively influences the overall filtration performance^1,2^. It has a pivotal role in ensuring the mechanical support to the membrane surface and help the fluid flow between the membrane sheets while aiding in minimizing the concentration polarization/(bio)fouling by promoting fluid unsteadiness^2–4^ inside the filtration channel^5,6^. However, inappropriate spacer designs are known to aggravate (bio)fouling and ultimately increase channel pressure drop, resulting in deteriorating the performance of the filtration system^3,7–10^. The formation of biofilm, which is the proliferation of bacteria within extracellular polymeric substances (EPS) matrix, is reputed to be so far the inevitable impediment in water technologies^11,12^. Therefore, the ability of biofilm mitigation constitutes a crucial trait to consider for designing a pertinent feed spacer. A maximal mass transfer while simultaneously reducing pressure drop and (bio)fouling development is then the ultimate engineering challenge to design an optimal feed spacer for different filtration techniques.

In the past, research efforts were focused on modestly modifying the geometric parameters of commercial spacer such as spacer thickness, filament diameter, mesh size, internal filament angle, and spacer orientation to amend the hydrodynamics conditions within the feed channel^8,13–19^. The commercial spacer design, which consists of two layers of non-woven quasi-cylindrical filaments, creates a narrow spacer-filled channel (low porosity) that intensifies the pressure drop^17,18^. Moreover, asymmetric spacer filament intersections created due to non-woven design promote local dead zones^20,21^, which are recognized as favorable areas for (bio)fouling growth^22^. An intricate hydrodynamics nature is further identified due to unsymmetrical shear stress distribution on either side of the membrane surface along the non-uniform filaments. Also, the inability to trip hydrodynamics to unsteady state (near plant operational cross-flow velocities of 0.165 m/s^23^) adds fundamental limitations for the commercial spacer designs^9^.

Attempts have been concentrated on developing novel feed spacer designs to eliminate the drawbacks of the commercial type spacer design^4,22,24–28^. Multilayer spacers revealed a more significant mass transfer and long-term anti-fouling propensity compared to a net-type spacer^25,28^. Triangular filament spacer shape was found to outperform square and circular filament spacers in terms of concentration polarization reduction^24^. Symmetric spacer with spherical nodes (filament intersections) demonstrated its capacity to produce high shear stress distribution and mass transfer rate^22^. A static mixing spacer showed an enhanced mass transfer performance than a conventional design at low range of flow rates^29^. Ladder and herringbones-shaped spacers were invented by Shrivastava et al. in the aim of studying the impact of geometric spacer design on reducing the concentration polarization on membrane surface^30^. More complex spacer designs like honeycomb-shaped spacer^31^, triply periodic minimal surfaces (TMPS)^26^, vibrating spacers^27^, turbo-spacer using rotating turbines^32^, and helical-type spacers^4^ have shown promise in reverse osmosis (RO) and ultrafiltration (UF) processes. Although these proposed novel spacer designs indicate favorable progress for enhanced filtration membranes, several constraints are still encountered and hamper their commercialization and industrial implementation. It includes the design complexity^26,32^, the handling difficulty^27,32^, the risk of membrane deformation/damage^24,25,33^, and the high energy requirements^22,25,28^.

Recently, promising novel spacers have been developed by our research group and have shown an attractive industrial interest for scale-up^21,34,35^. These feed spacers are characterized by the introduction of perforations (perforated spacers)^34,35^ or column nodes (column-type spacer)^21^ in their designs. Their performances were numerically and experimentally evaluated in a cross-flow UF process. The most efficient design was demonstrated with spacer having perforations only at the filament intersections^34^. Perforated spacer design helped to lower the pressure drop and induce more unsteadiness within the filtration channel, resulting in enhanced permeate flux production and fouling mitigation. Likewise, the column-type feed spacer led to simultaneously reduce three times the pressure drop, increase two times the specific flux, and minimize the fouling accumulation when compared to the standard symmetric spacer^21^.

The present study was inspired by the encouraging outcomes achieved by the perforated and column (referred to as pillar hereafter) spacers^21,34^. The objective was to combine their advantages and geometric features to develop a novel feed spacer design for enhanced filtration processes, which is easily manufactured in an industrial scale. Consequently, symmetric pillar feed spacer with perforations (hole-pillar spacer) is introduced as a novel design. The proposed design can induce higher channel porosity, control localized hydrodynamics, and evenly distribute the shear stress inside the filtration channel. All these design features are aimed to improve the filtration performance, reduce the pressure drop, and mitigate the (bio)fouling accumulation on the membrane surface, which is an ultimate challenge for feed spacer design used in different filtration technologies including UF, nanofiltration (NF), and RO.

Three feed spacers were in-house designed and 3D-printed: commercial, pillar, and hole-pillar spacers. We first comprehensively evaluated these spacer designs by direct numerical simulations (DNS) to study the localized hydrodynamics. Later, the effect of spacer design (commercial, pillar, and hole-pillar spacers) on the filtration performance including pressure drop measurement, permeate flux analysis, and Optical Coherence Tomography (OCT) characterization for biofilm growth, is experimentally investigated in UF cross-flow filtration for biologically active feed at two different operating pressures. Finally, potential outcomes from this study and its perspectives are discussed.

## Methods and materials

### Feed spacer designs and numerical formulation

**Figure 1:**
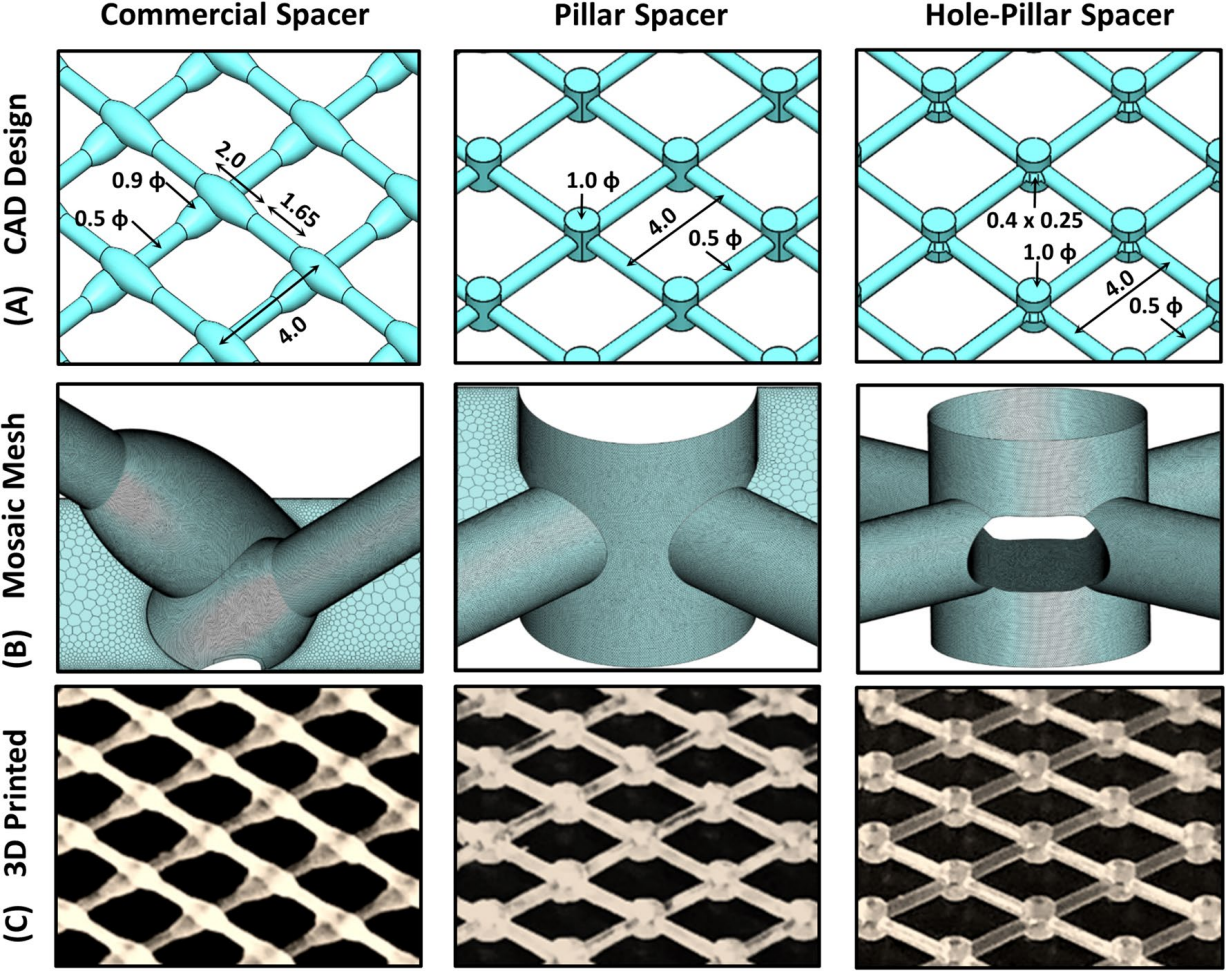
Design of the novel hole-pillar spacer along with commercial and pillar spacer designs used in the present work. (**A**) Computer-Aided Design (CAD) along with relevant dimensions, (**B**) Zoomed view of ANSYS Mosaic mesh generation utilized for direct numerical simulations (DNS), and (**C**) 3D-printed spacers. All spacers are aligned 45° with respect to the incoming feed flow. Respective dimensions are in mm and the thickness of all spacers is H = 1.2 mm.

Three feed spacers, namely commercial, pillar, and hole-pillar (perforated in filament intersections) spacers were designed and generated using Computer-Aided Design (CAD) on *SolidWorks* software (Dassault Systemes SolidWorks Corporation, Waltham, MA, USA, Version 2018). For commercial spacer design, a 34 mil spacer design (Dow Chemical Company, MI, USA) was scaled as per SEM dimensions reported by Radu et al.^14^. The feed spacers were manufactured by using a high-resolution 3D-printing technology (MiiCraft 125, Version 3.4.5, MiiCraft, Germany) for rapid prototyping. Figure 1 shows the three spacer designs along with their relevant dimensions.

The numerical calculations were performed using DNS approach, allowing accurate capturing of relevant flow scales without any additional turbulent models. The full mathematical implementation details are presented in our previous work^36^. The details of the computational domain and boundary conditions are provided in the supplementary material (Fig. S1). For the current simulations, an advanced ANSYS Mosaic^37^ grid generation technology was utilized. These mesh grids wrap the CAD body with isotropic poly-prism and translate to hexahedral elements in the fluid volume regions. Figure 1 shows the mesh generation on the surface of the feed spacer. All relevant corners are captured quite well with appropriate length scales to resolve the incoming flow. Mesh independence studies were performed before the simulations (Table S1, in supplementary material) and the final mesh of ~ 26 million grid points was found sufficient to resolve the local hydrodynamics. The mesh independence study was performed prior to numerical investigation and a relevant mesh of ~ 26 million grid points was found sufficient to resolve the flow field.

The solution of the system of Navier–Stokes equations^36^, along with the specified boundary conditions (supplementary material, Fig. S1), was performed in a commercial solver ANSYS Fluent (ANSYS, Inc., R2-2020) that utilizes the finite-volume approach^38,39^. A second order implicit formulation for temporal discretization was utilized. The pressure term was also computed using second order accuracy, while the momentum term followed the QUICK (Quadratic Upstream Interpolation for Convective Kinematics)^39^ formulation using ANSYS Fluent default relaxation factors. PISO scheme^38^ was used to maintain pressure–velocity coupling during the computations.

### Feed solution preparation

A feed solution of 4 L of Red Sea water containing 4 g of Bacto Yeast Extract (extract of autolysed yeast cells, Becton, Dickinson and Company) to aggravate biofilm growth was utilized for all conducted UF experiments^9^. In more detail, 4 g of Yeast Extract was dissolved in 1 L of real Red Sea water and incubated at 30 °C for 24 h to enhance the bacterial proliferation in the solution. Then, the incubated solution was diluted by adding 3 L of real Red Sea water^40^ and continuously stirred at 200 RPM with a magnetic stirrer (IKA, model n° RCTBS002) throughout UF experiments. The feed solution was continuously adjusted during the filtration process by a fresh solution of Yeast Extract prepared in Red Sea water (1 g/L) to maintain a total volume of 4 L.

### Experimental protocol for UF tests

**Figure 2:**
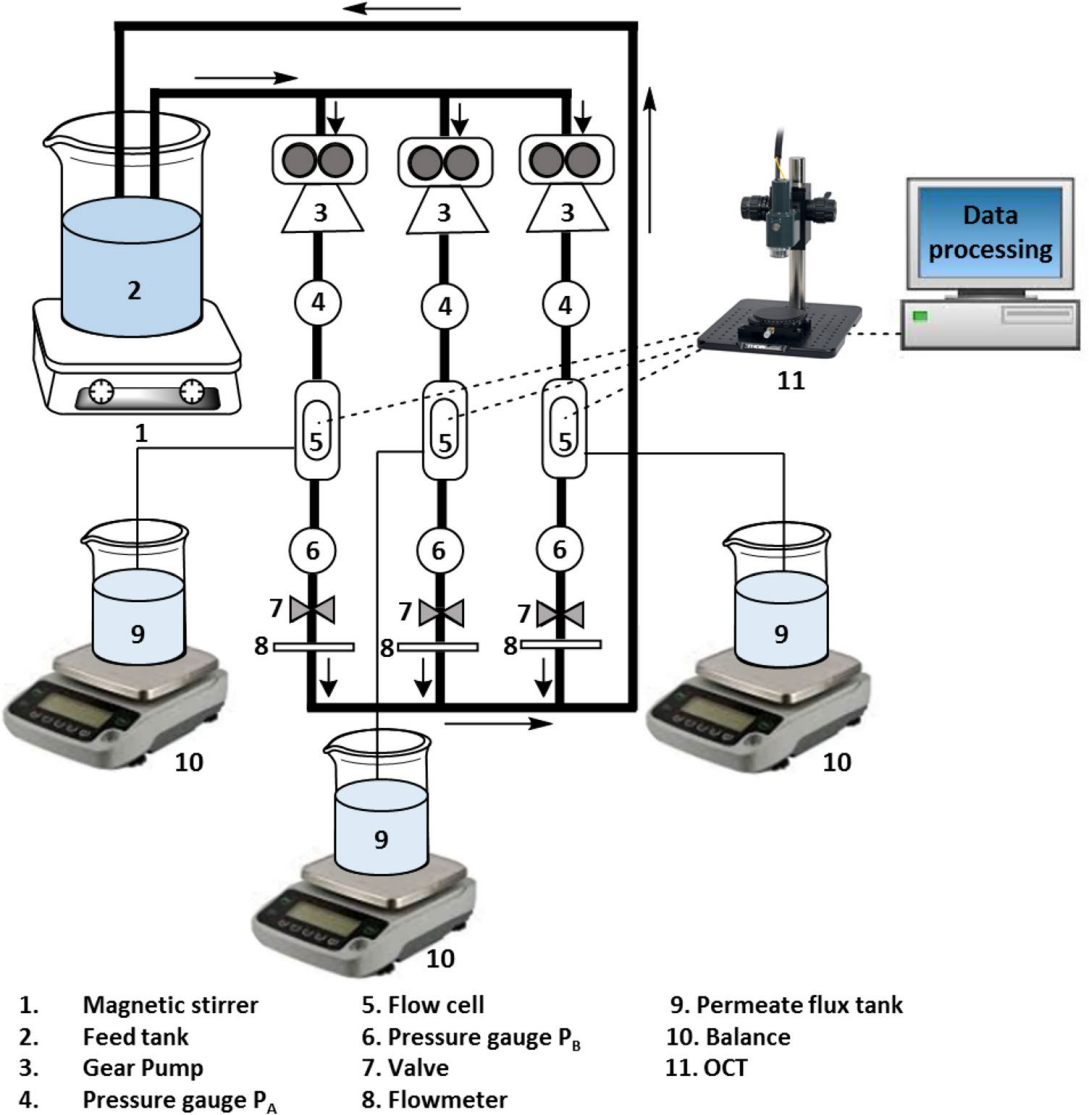
Schematics describing the lab-scale setup and the various instruments utilized for the lab-scale UF experiments. All experiments were performed for 67 h that were sufficient for all spacers to reach the steady-state condition (stable permeate flux) of filtration.

Three cross-flow UF experiments were carried out simultaneously (in parallel) for the three tested spacers at two different operating pressures (P = 0.5 and 1.0 bar). The setup and various equipment used are schematically described in Fig. 2. The same batch of prepared feed solution was pumped to three identical flow cells by three similar gear pumps (Cole-Parmer, model n° 75211-70, head: N23) and then recirculated back to the feed tank. The feed filtration channel of each flow cell had a height of 1.2 mm. The feed spacer was then 3D-printed with a height of 1.2 mm to match the feed channel depth and conduct UF processes in a spacer-filled filtration channel. Therefore, the spacer height was defined to fill the distance between the membrane surface and the acrylic glass of the flow cell feed channel. The feed channel is composed of UF membrane (flat sheet Sterlitech membrane, polyethersulfone, molecular weight cut-off of 100 kDa) and spacer coupons, which were cut to fit the filtration channel dimensions (60 mm × 15 mm). For each filtration module line, two pressure gauges (Ashcroft Inc., model n° 1005) were installed at the input and output of the flow cells. 0.5 bar and 1.0 bar hydraulic pressures were applied throughout the entire UF process (67 h). At the same time, a volumetric flow rate of 200 mL/min (inlet feed velocity U_o_ = 0.185 m/s) was monitored by using a flowmeter (Dwyer, model n° RMB-SSV) placed at the output side. Desired pressure and flow rate inside the flow cell were controlled by using a valve located before the flowmeter. The weight of permeate flux for each filtration module was measured using a separate digital balance (Mettler-Toledo, model n° MS3002S) and recorded automatically via a data acquisition system (National Instruments, LabVIEW, 2016). The biofouling visualization was achieved by a two-dimensional (2D) Optical Coherence Tomography (OCT) (Thorlabs, Hyperion) taken on membrane surface perpendicularly to the flow direction at 16 h and 67 h of UF experiments and then post-processed by utilizing *ImageJ* software (U.S. National Institute of Health, Version 1.48).

Before filtration experiments, the pressure drop of each spacer was independently evaluated in a cross-flow setup (similar to Fig. 2) with the same feed solution at various inlet cross-flow velocities. A differential pressure transmitter (Omega Engineering, model n° PX5200 M5091/0112) was deployed to accurately measure the initial pressure drop associated with each spacer design in the filtration channel.

## Results and discussion

Fundamental hydrodynamics characterization, UF performance investigation, and in-situ biofilm observation of the novel hole-pillar spacer were performed under varying applied pressure conditions (0.5 bar and 1.0 bar) for a fixed inlet flow channel velocity of U_o_ = 0.185 m/s. For comparative purposes, the performance of the hole-pillar spacer was compared with the commercial and pillar type spacers without holes. The hydrodynamics of each spacer design was first studied at an elemental level through numerical simulations achieved by DNS. Subsequently, UF experiments were carried out to investigate the flux performance under various applied pressures, along with in-situ biofilm monitoring of each spacer by utilizing the OCT technique.

### Numerical simulations

DNS simulations were performed for the three tested spacers at an inlet flow velocity corresponding to the experimental conditions (U_o_ = 0.185 m/s). The pressure drop gradients, local velocity magnitudes, and shear stress distributions inside the filtration channel of various spacers are presented in this section.

#### Pressure drop gradients and validation of DNS model

The pressure drop over the spacer length is a significant trait of the spacer design and configuration, deciding the overall performance of the filtration module. In particular, the channel pressure drop across the module is directly related to the total energy consumption of the filtration process. As filtration progresses, a poor spacer design would increase (bio)fouling or concentration polarization, increase the channel pressure drop, and effectively tarnish the performance of the filtration module.

**Figure 3:**
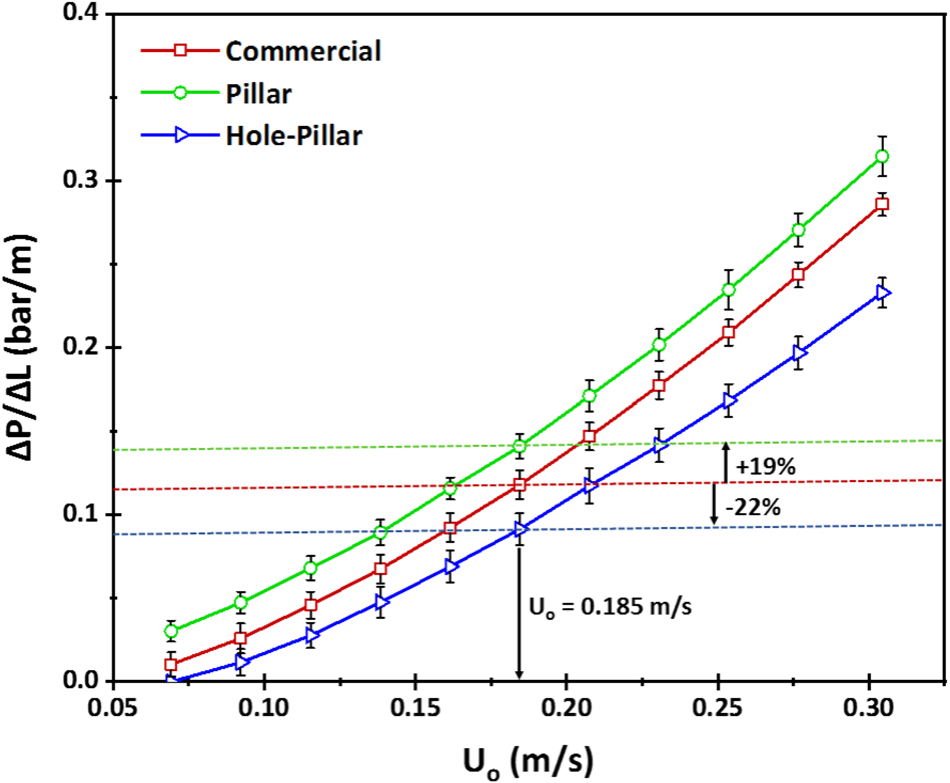
Experimental pressure drop gradients along the filtration channel length as a function of various inlet velocities for the three tested spacers. The lowest pressure drop is observed for hole-pillar spacer, followed by commercial and then pillar spacers.

Figure 3 shows the experimental pressure drop gradients ($\Delta P/\Delta L$) across the filtration channel at various inlet channel velocities. These measurements were performed before UF experiments, intuitively indicating which design would have better energy efficiency. Apart from that, it also serves to validate data for the performed DNS calculations. Pressure drop gradients were measured at an inlet velocity ranging between 0.07–0.3 m/s. For all the spacer designs, a non-linear rise roughly close to quadratic behavior was observed. The pillar spacer design produced the highest pressure drop gradients corresponding to all flow velocities. At filtration experimental conditions (U_o_ = 0.185 m/s), the pressure drop gradients were approximately 0.118, 0.141, and 0.091 bar/m for commercial, pillar and hole-pillar spacers, respectively. An increase of 19% of pressure drop gradient was obtained for the pillar spacer relative to the commercial spacer, whereas a reduced pressure drop gradient of 22% was achieved for the hole-pillar spacer. The obtained experimental results were compared with the pressure drop gradients computed by DNS calculations, as presented in Table 1. The numerical results were found to have close values to the experimental results with an error range less than 11%, indicating as well that the hole-pillar spacer has the least pressure drop gradient, followed by the commercial and then pillar spacers.

**Table 1:** Values of experimental pressure drop gradients at inlet velocity of Uo = 0.185 m/s compared with DNS computations for solver validation.

Feed spacer	Experimental ΔP/ΔL (bar/m)	Numerical ΔP/ΔL (bar/m)	Error between experimental and numerical ΔP/ΔL (%)	ε
Commercial	0.118 ± 0.009	0.128	− 8%	0.89
Pillar	0.141 ± 0.007	0.134	5%	0.88
Hole-Pillar	0.091 ± 0.009	0.101	− 11%	0.90

ε represents the calculated channel porosity. The percentage errors between the experimental and numerical pressure drop gradient values are calculated relative to the experimental values.

The present comparison between experimental and numerical pressure drop gradients establishes the validity of the used mathematical model (Table 1). The low percentage error between experimental and numerical values (below 11%) is primarily attributed to slight variation in spacer dimensions during 3D-printing, meshing of complex spacer designs, and fluctuations in sensor and other equipment involved in the experiments. Besides, the pressure drop gradients were found to be sensitive to the channel porosity (ε). The lowest porosity was found for pillar spacer (0.88), followed by the commercial spacer (0.89), and then hole-pillar spacer (0.90) which owns the lowest pressure drop. Although pressure gradient trends were observed following the channel porosity, it was also associated with the local hydrodynamics of each spacer design (as discussed in the subsequent section). This suggests that to effectively design an optimal novel spacer with low pressure drop, an attempt should be made to maximize the channel porosity and optimize as well the local hydrodynamics to reduce the initial energy requirements of the filtration process.

#### Local flow field velocity for various spacers

Local hydrodynamics occurring within the single spacer cell is essential to comprehend the physical insight into its performance evaluation. Higher local velocity near the membrane surface is essential to minimize concentration polarization, while it is detrimental if the feed is biologically active as it has the potential to aggravate biofilm development (high shear induces faster biofouling development^41,42^), especially in desalination pre-treatment processes like UF and NF. Consequently, it is essential to interpret local hydrodynamics for each spacer design to gauge potential performance.

**Figure 4:**
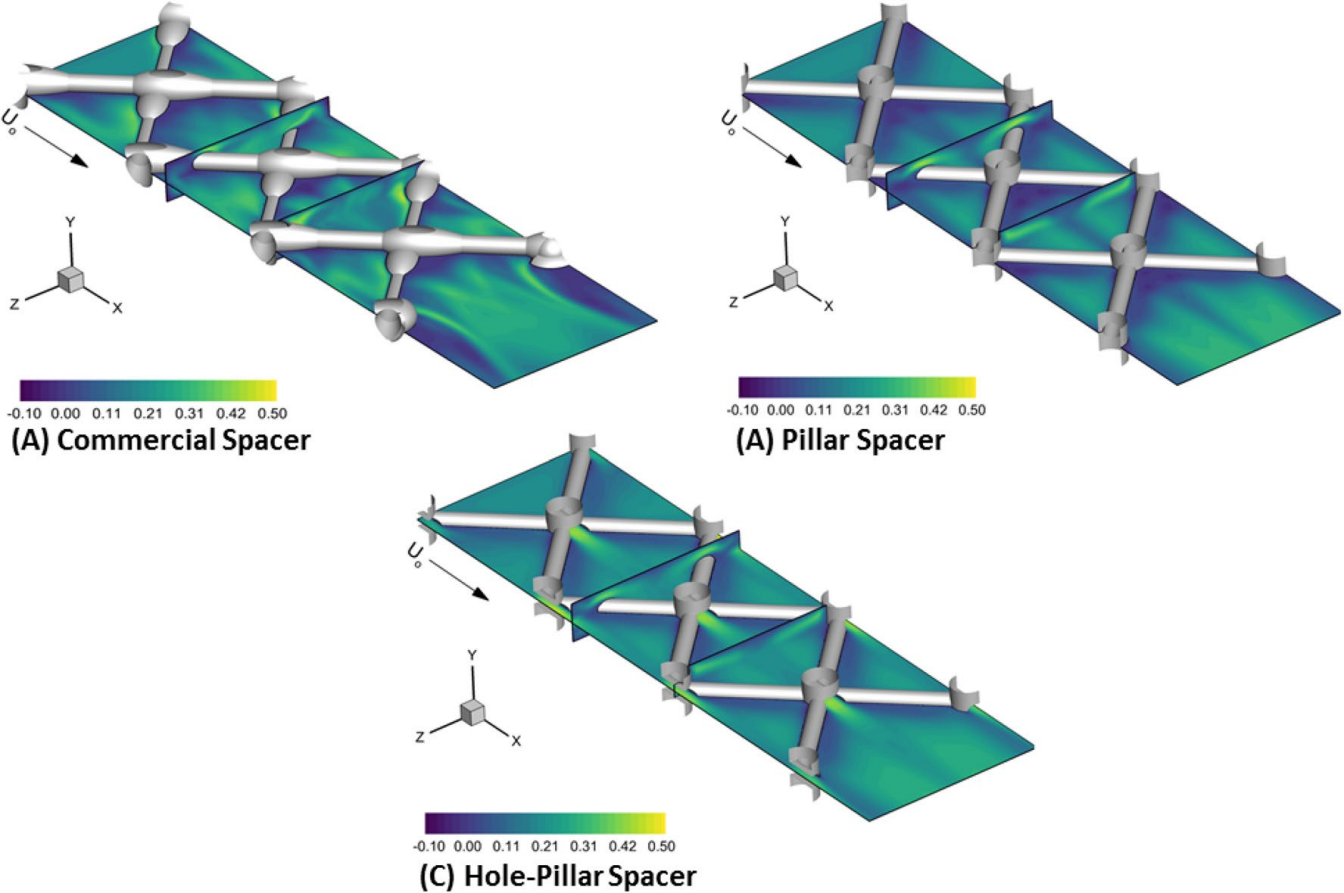
Local flow x-velocity magnitudes using DNS computations at different slice locations for the three spacer designs at U_o_ = 0.185 m/s used in actual filtration experiments. The dark violet color indicates flow separation areas (dead zones) as seen by negative x-velocity magnitude. Micro-jet formation visible behind the pillar of hole-pillar spacer is effectively enhancing filtration performance and reducing pressure drop.

Figure 4 shows the DNS contours of x-velocity component at various planes inside the computational domain for the three spacer designs at an inlet velocity of U_o_ = 0.185 m/s. The x-velocity is the velocity component along the flow direction. It can clearly represent areas where velocity is negative (dark violet color), indicating the formation of recirculation regions (dead zones)^20,21^. At U_o_ = 0.185 m/s, all spacer designs settle to steady hydrodynamics, suggesting that no-fluctuations in the localized velocity field are present. The u-velocity magnitude picks up to satisfy the local mass and momentum conservation under the spacer filament, which is proportional to the clearance region (space between the spacer filament and the membrane surface)^9^. Being non-woven in design, the commercial spacer produces asymmetric flow field (Fig. 4A). As the commercial spacer filament cross-section along the filament length is not uniform (Fig. 1), the clearance is least on the thickest side of the filament (clearance ≈ 0.15 mm) and it produces the highest velocity (≈ 0.46–0.50 m/s) on the membrane surface. However, the velocity of the thinnest side of commercial spacer filament having a highest clearance (≈ 0.35 mm) is approximatively estimated to be 0.42 m/s.

On the other hand, the pillar and hole-pillar spacers produce a symmetric flow velocity pattern around their filaments as the clearance height (≈ 0.35 mm) is maintained along the filament of both spacers (Fig. 4B,C). The local x-velocity magnitude on the membrane surface is lower (≈ 0.36–0.42 m/s) under their filaments compared to the thickest side (lowest clearance) of the commercial spacer filament (≈ 0.46–0.50 m/s). The hole-pillar spacer has the least x-velocity magnitude under the filament (≈ 0.36 m/s). This is attributed to the distribution of incoming feed flow momentum being distributed between the filament clearance and pillar hole to produce a local jet inside the filament cell.

**Figure 5:**
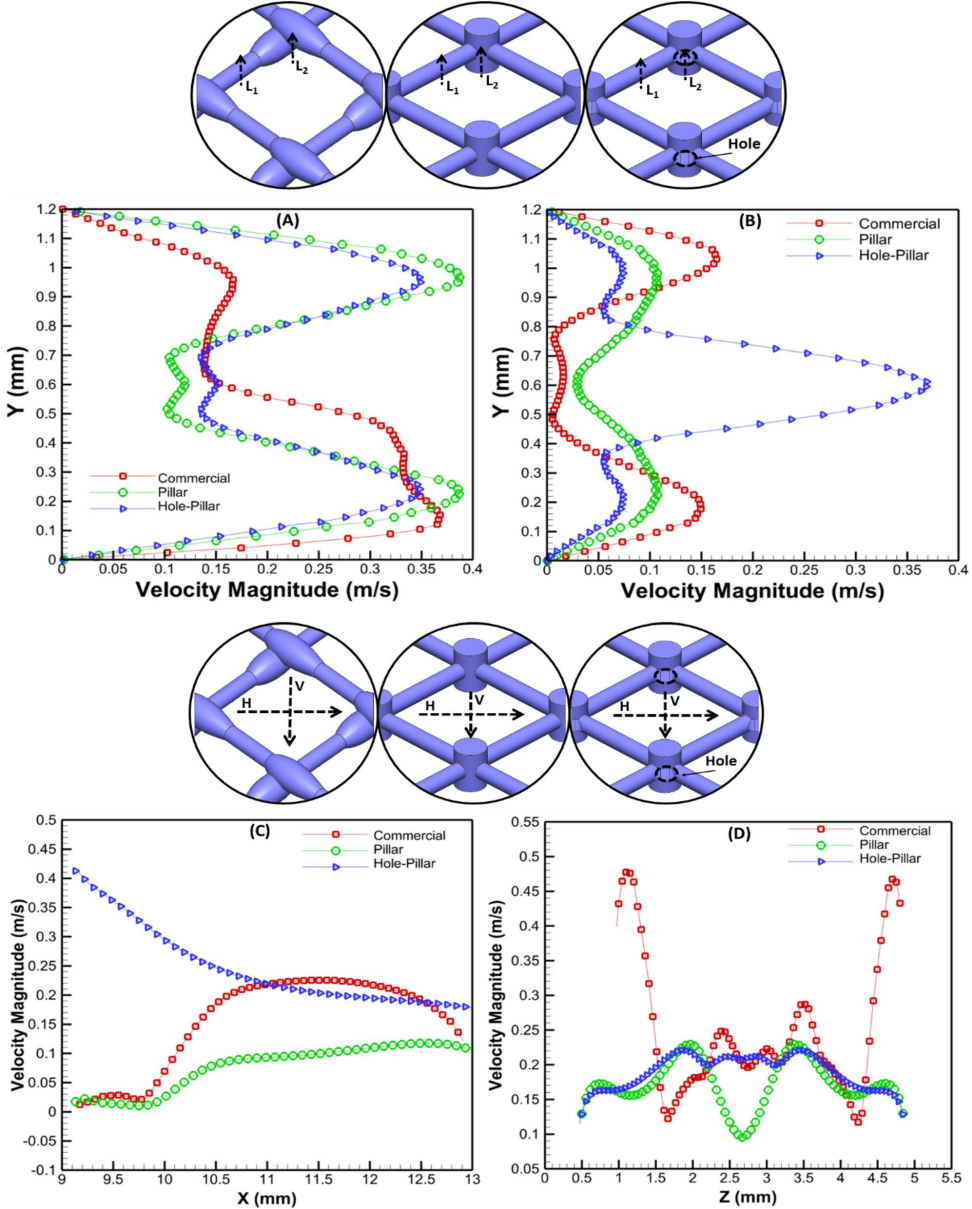
Velocity magnitude profiles computed using DNS computations at different locations: (**A**) Line L_1_ (from the bottom wall of the filtration channel to the top wall), (**B**) Line L_2_ (from the bottom wall of the filtration channel to the top wall), (**C**) Line H (from the left of the unit flow cell to the right), and (**D**) Line V (from the top wall of the channel to the bottom wall) for the three spacer designs at U_o_ = 0.185 m/s. Boundary layer profiles (L_1_ and L_2_) and spatial profiles (V and H) clearly indicate formation of jets and higher local velocities achieved inside the spacer filament for the hole-pillar spacer compared to the commercial and pillar spacers.

The localized hydrodynamics is further studied by evaluating the boundary layer profiles (locations L_1_ and L_2_) behind the filament and filament intersection of each spacer design, and along the horizontal (location H) and vertical (location V) lines inside the spacer cell, as depicted in Fig. 5. Figure 5A (location L_1_) indicates clearly that the asymmetric nature of the boundary layer profiles is seen for the commercial spacer, while those of the pillar and hole-pillar spacers are perfectly symmetric as seen in cylinder flow dynamics^43^. However, the average profile values calculated along the channel height are roughly similar for all spacers. Near the filament intersection (location L_2_—Fig. 5B), the boundary layer profiles clearly indicate the velocity magnitude increase for the hole-pillar spacer compared to the commercial or pillar spacers. In general, this region of the spacers is potentially prone to particle accumulation due to the formation of recirculating zones. The hole-pillar spacer effectively eliminates this fundamental drawback by inherently forming a fluid jet due to its unique design. Further, along the horizontal line (location H—Fig. 5C), the velocity magnitude of the hole-pillar spacer is relatively higher until half-length of the spacer cell (X ≈ 10.9 mm) and ultimately converges to values of commercial and pillar spacers for the rest of the spacer cell unit, suggesting effective cleaning performance by the hole created at the pillar intersection. On the other side, along the vertical length (location V—Fig. 5D), the commercial spacer appears to have the highest velocity toward the end (lowest clearance available due to increasing filament diameter at intersection). However, the rise in velocity is in a narrow region, and it quickly falls in the velocity magnitude range of the pillar and hole-pillar type spacers.

The hydrodynamics performance clearly indicates that commercial spacer has a tendency of high asymmetric one-sided local velocity, while the pillar spacer produces similar level velocity magnitude under the filament in a symmetric pattern. The hole-pillar spacer has the potential to not only maintaining symmetric hydrodynamics under the filament but also producing higher velocity behind the filament intersection, which aids to improve the hydrodynamics conditions for enhanced filtration performance.

#### Shear stress distribution for various spacers

Shear stress distribution generated on the membrane surface by various spacer designs and hydrodynamics conditions is a critical component that dictates biofouling characteristics in a filtration system. A quick bacterial attachment on membrane surface occurs at high shear stress regions, followed by bacterial colonization and finally biofilm expansion and migration into low shear regions^9^. Contrary, the concentration polarization is minimized if the shear stress is higher on the membrane surface, while it is higher in low shear regions^16,22^. Consequently, designing a universal spacer is quite challenging as shear stress on the membrane surface has to be tailored according to the desirable filtration need or technique.

**Figure 6:**
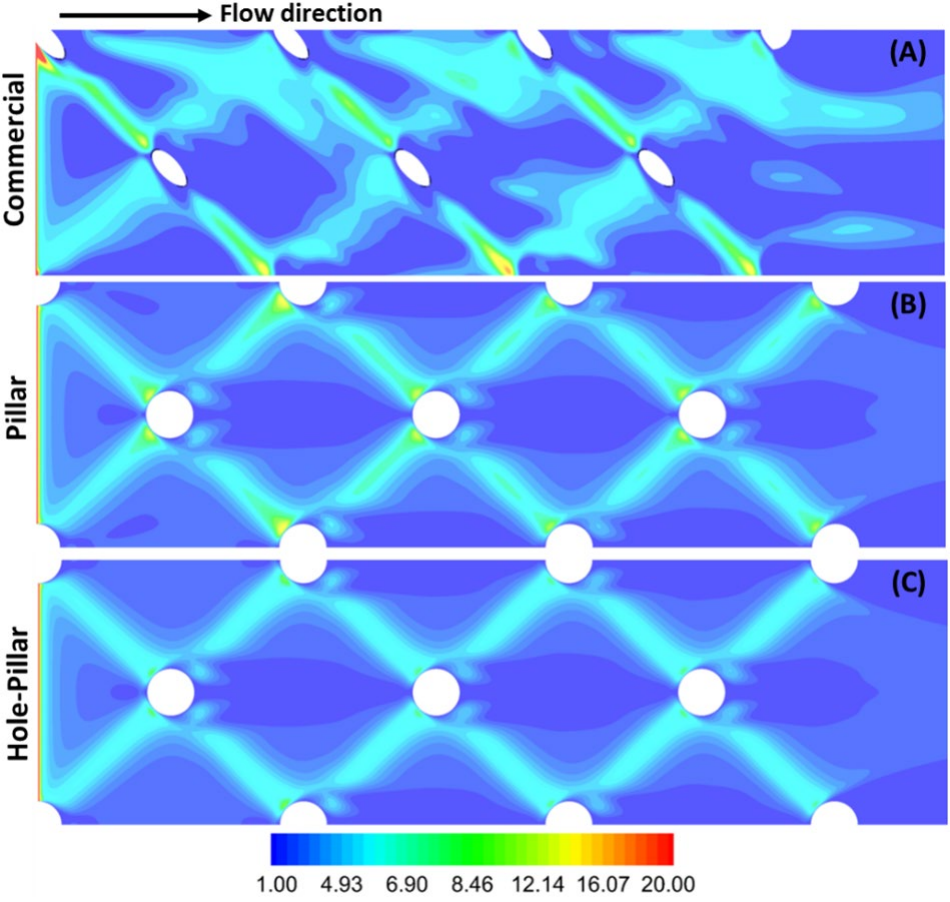
Calculated shear stress distribution on the bottom wall of the filtration channel. The commercial spacer has asymmetric stress distribution with the highest shear stress under the filament. Pillar and hole-pillar spacer have symmetric stress distribution, and lower shear stress value is observed for the hole-pillar spacer.

Figure 6 shows the shear stress distribution for the three spacer designs at an inlet flow velocity of U_o_ = 0.185 m/s. The shear stress contours are evenly distributed for the pillar and hole-pillar spacers compared to the commercial design. For all spacers, the highest shear is found under the filaments presented obstructions against the fluid flow. Due to non-woven asymmetric design and one-sided low clearance in the commercial spacer (thick filament side), it produces the highest average shear values (≈ 12–16 N/m^2^) under the filament. While for the pillar spacer, the local fluid momentum is slightly reduced due to the symmetric alignment of spacer filament, leading to lower average shear values (≈ 12–13 N/m^2^) under the filament. In contrast, the hole-pillar spacer produces very even, and the lowest average shear stress (≈ 5–6 N/m^2^) as the hole at the pillar intersection redistributes the local fluid momentum.

The shear stress distribution associated with hydrodynamics behavior allows predicting the suitability of a spacer design for the desired filtration technology. Among the three spacer designs, it is clear that commercial spacer would perform ideal for minimizing concentration polarization (like seen in RO applications where in general the feed is devoid of bacteria by pre-treatment process) as it has higher local velocity/shear stress and allows asymmetric distribution for feed flow. On the other hand, the pillar and hole-pillar spacers are symmetric in design with constant filament cross-section diameter. As demonstrated above, this spacer filament type aids in producing even fluid velocity distribution, associated with low shear stress on the membrane surface resulting in better mitigation of biofouling growth. Consequently, these spacers are mainly dedicated for filtration processes in which biological active feeds are utilized. Thus, pillar and hole-pillar spacers are ideal candidates for filtration of raw water feeds rich in active bacterial components, such as UF, NF, and low-pressure RO systems used for surface water treatment or as desalination pre-treatment for RO. The present DNS study provides an excellent approach to evaluate spacer design and potential performance.

### Experimental flux performance under varying applied pressure

**Figure 7:**
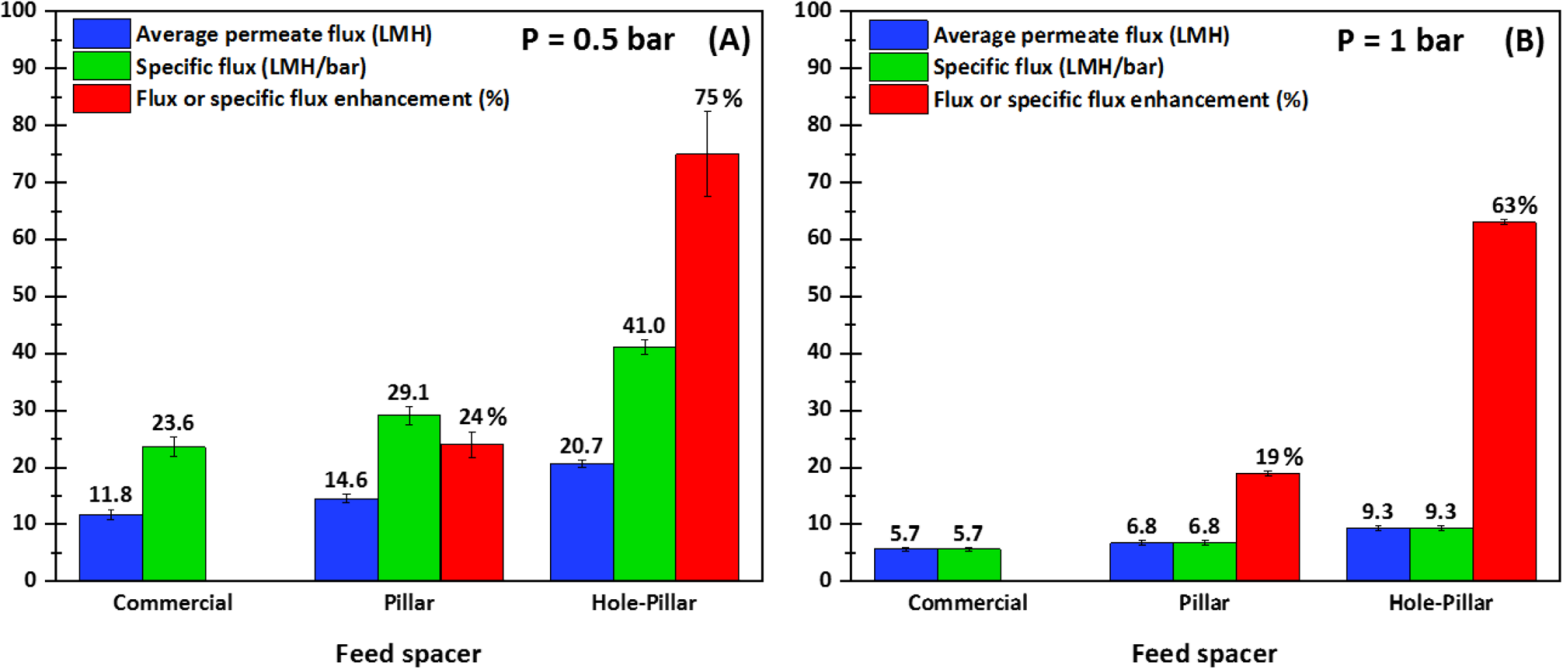
UF filtration performances at steady-state after 67 h of filtration process in presence of various tested spacers for two applied pressures: (**A**) P = 0.5 bar, and (**B**) P = 1.0 bar. The specific flux is determined relative to transmembrane pressure. The flux and specific flux enhancement were calculated relative to the commercial spacer.

The three spacer designs were experimentally tested in a cross-flow UF channel to measure the performance of the hole-pillar spacer compared to the commercial and pillar spacers. For all conducted tests, the cross-flow velocity was fixed at U_o_ = 0.185 m/s and two pressures of 0.5 and 1.0 bar were applied for each spacer using biologically active feed. Figure 7 shows the average permeate flux obtained at steady-state conditions (when the flux becomes stable) after 67 h of UF process along with the specific fluxes for all tested spacers. The specific flux is defined as the permeate flux produced in filtration process per unit of transmembrane pressure. Therefore, the determination of specific flux is vital to assess the overall performance of spacer design in terms of filtration productivity and energy consumption.

Regardless of the applied pressure, the highest average flux is observed for the hole-pillar spacer, followed by the pillar and commercial spacers, respectively. At an applied pressure of 0.5 bar, the hole-pillar spacer produced 20.7 LMH, while the pillar and commercial spacers produced 14.6 LMH and 11.8 LMH, respectively. Based on the initial flux values, the percentages of flux declines were calculated to be 85%, 84%, and 77% for commercial, pillar, and hole-pillar spacers, respectively. This finding confirmed that the hole-pillar spacer outperformed the other spacers in terms of flux production. When the applied pressure was increased to 1.0 bar, the permeate flux trend remained the same for the different spacer designs (9.3 LMH, 6.8 LMH, and 5.7 LMH for the hole-pillar, pillar, and commercial spacers, respectively). However, the permeate fluxes produced at P = 1.0 bar are found significantly reduced in a percentage range of 50–55% for all spacers compared to those obtained at P = 0.5 bar. Furthermore, the flux decline percentages for all spacers, which were estimated at 93% for commercial and pillar spacers and 91% for hole-pillar spacer, were found to be higher at higher pressure. This trend is primarily attributed to the growth of biofilm under varying operating conditions, which revealed faster bacterial development on membrane surface at higher applied transmembrane pressure (discussed subsequently in Section “Biofilm characterization by OCT”).

Likewise, similar trends are observed for the specific flux values. The highest specific flux is observed for hole-pillar spacer (41 and 9.3 LMH/bar at P = 0.5 and 1.0 bar, respectively) followed by pillar spacer (29.1 and 6.8 LMH/bar at P = 0.5 and 1.0 bar, respectively), and least for commercial spacer (23.6 and 5.7 LMH/bar at P = 0.5 and 1.0 bar, respectively). Similarly to permeate flux behavior, the specific flux values are reduced for all spacers in a percentage of 77% when increasing the applied pressure from 0.5 to 1.0 bar. The percentage permeate flux (or specific flux) enhancement clearly suggests that the hole-pillar spacer outperforms the commercial spacer design by 75% and 63% for the two applied filtration pressures of 0.5 and 1.0 bar respectively, whereas the pillar spacer outperforms the commercial spacer by 24% and 19% for 0.5 and 1.0 bar respectively. These insights revealed that the application of low pressure on cross-flow UF channels equipped with these spacers is more effective for enhanced filtration performance and reduced energy consumption.

### Biofilm characterization by OCT

Understanding biofilm growth on the membrane surface for biologically active feeds is critical for filtration performance^44^. Not only the permeate flux output is affected by growing and collapsing biofilm, but also the energy requirements are significantly influenced by the biofilm development. As biofilm grows, it reduces the porosity of the filtration channel, resulting in an exponential rise in pressure drop inside the filtration channel. With severe biofouling, the increasing channel pressure rise has to be tackled by a feed pump, thereby consuming higher energy for maintaining the production of the flux^44^. The biofilm growth is primarily connected with feed spacer design and is critical to evaluate.

**Figure 8:**
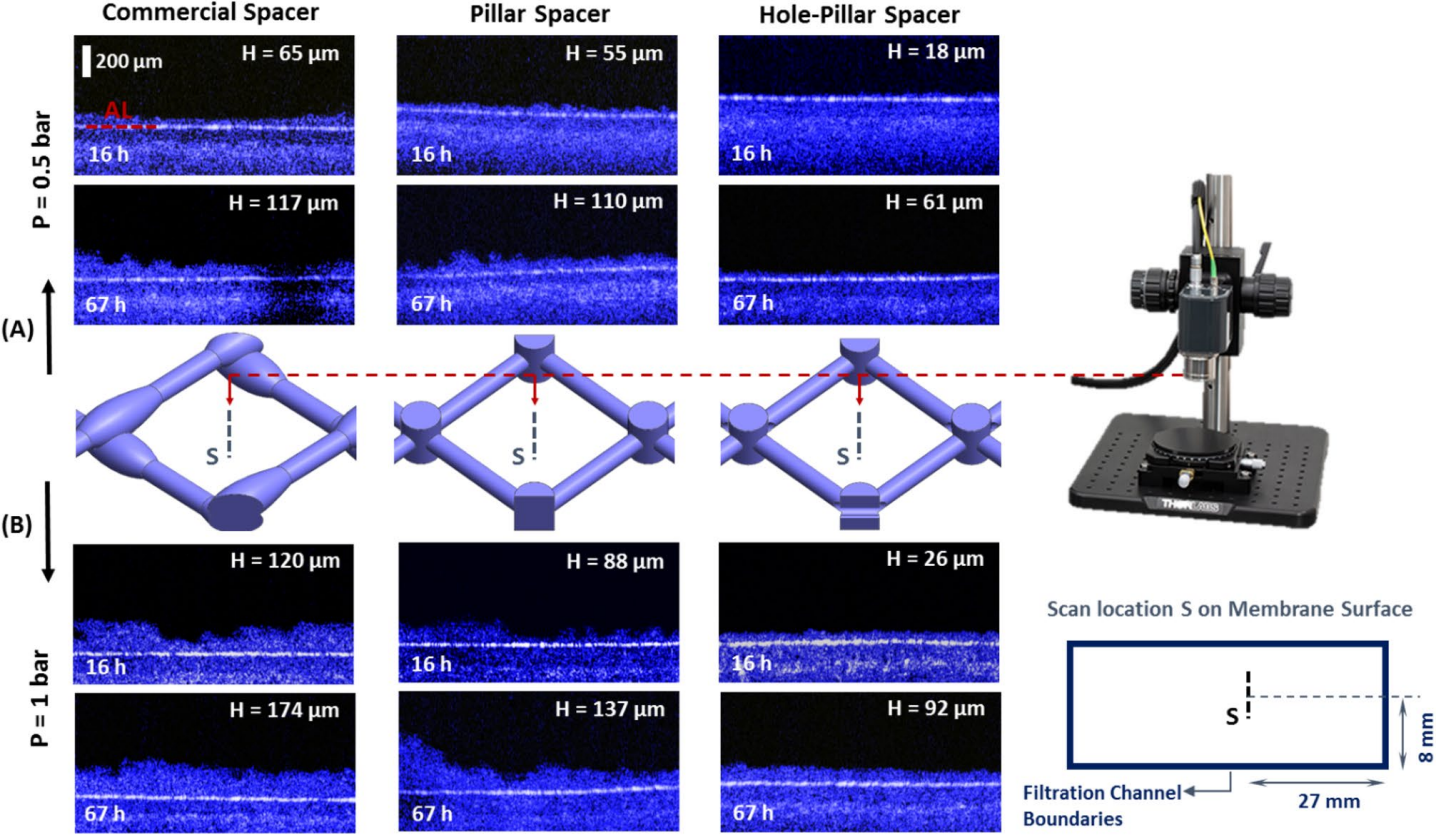
OCT scans taken at line S located at the center of the spacer cell. Biofilm variation on the membrane surface for the three tested feed spacers at applied pressures of P = 0.5 bar (**A**), and P = 1.0 bar (**B**) taken at 16 h and 67 h of filtration process. The scale bars of all images are the same. AL line represents the membrane active layer. Below AL is the membrane support layer and above is the deposited biofilm.

Figure 8 shows the OCT scans taken on the membrane surface at the spacer cell center for the three spacer designs at 16 h and 67 h of filtration process (additional OCT images taken at a location close to the spacer filament are shown in Fig. S2 of supplementary material). As the feed solution is the same for all spacer designs and the operating parameters (applied pressure and cross-flow velocity) are fixed, intrinsic biofilm development of each design can be characterized in a controlled manner. For filtration occurring at 0.5 bar, the OCT scans reveal that commercial spacer consistently tends to form a higher average biofilm thickness H (H = 65 μm at 16 h and 117 μm at 67 h, as shown in Fig. 8A (left)), followed by the pillar spacer (H = 55 μm at 16 h and 110 μm at 67 h, Fig. 8A (middle)), and the least biofilm thickness was observed for the hole-pillar spacer (H = 18 μm at 16 h and 61 μm at 67 h, Fig. 8A (right)). The trends are consistent with permeate flux performance (Fig. 7A). The cleaner is the membrane surface due to the appropriate spacer design, the higher is the permeate flux produced in presence of the corresponding spacer.

Similar to the low pressure counterpart, commercial spacer has the highest biofilm thickness (H = 174 μm at 67 h of filtration), followed by pillar spacer (H = 137 μm), and then by hole-pillar spacer (H = 92 μm) (Fig. 8B). Regardless of the filtration time and the spacer design, the biofilm thickness is found to be greater at high applied pressure (1.0 bar) compared to the biofilm developed at low pressure (0.5 bar) case.

The present outcome is unconventional as higher applied pressure usually results in higher permeation flux, especially for pre-treated/non-biological active feeds (like seen for RO operations). At higher applied pressure, organic or inorganic particles are forced to pass through the membrane structure. Thus, under this condition, it is anticipated that pore-blocking could occur which leads to a greater accumulation of bacteria and other foulants on the membrane surface^45,46^. Moreover, the bacterial attachment would also be greater as the normal shear stress (equivalent to static pressure^47^) is increased, resulting in higher biofilm thickness, as evident in the OCT scans.

Thus, the hypothesis discussed in Section “Shear stress distribution for various spacers” is confirmed. The commercial spacer that generated the highest shear stress on membrane surface resulted in the thickest biofilm cake. However, hole-pillar spacer helped to significantly mitigate the biofilm development on membrane surface due to its novel design which promoted lower shear stress within the filtration channel.

### Perspectives

The role of spacer design and the importance of operational parameters (mainly applied pressure) on UF filtration performance are clearly established in the present work. In general, feed spacer design should improve the filtration performance by gauging three design aspects. Firstly, the design should minimize the concentration polarization, resulting in improving the mass transport characteristic^16^. Secondly, it should produce high shear stress on the membrane surface to minimize particulate accumulation^48^. And lastly, the induced pressure drop due to spacer design should be minimal to reduce the energy requirements of the filtration process^16^.

For biologically active feeds, the striking feature that controls the filtration performance is the shear stress distribution on the membrane surface. As seen in the present work, the DNS calculations predict the highest shear stress for the commercial spacer, followed by the pillar and hole-pillar spacers. Clearly, from OCT images, the commercial spacer has the highest biofouling developed on UF membrane surface among the three spacer designs. This is contrary to the design of the feed spacer seen in RO systems, where high shear stress is better suited^49,50^. This indication is further reinforced if the comparison is made between the pillar spacer and hole-pillar spacer designs. As the hole-pillar spacer produces the least shear and even distribution of shear stress on the membrane surface, it results in the least biofouling. Thus, it is elucidated that for pre-treatment processes or applications where feed is biologically active, the shear stress on the membrane surface should be minimized to reduce the biofouling tendency. This finding is concurrent with biological studies where high shear is demonstrated to produce more significant biofilm attachment and growth on the surfaces/substrates^41,42^.

In addition to shear stress, interpretation of initial pressure drop across the channel is also confounded, especially for biologically active feed. For pre-treated feed, the low initial pressure drop is a good indicator for low energy expenditure as it clearly connected with the energy consumption of the pump. However, for non-treated feed, the initial pressure drop associated with spacer design is not a full indicator of the energy requirements. The initial pressure drop of a given design might be lower, but if it has the potential to produce higher shear stress on the membrane surface, then biofilm will be more pronounced in these scenarios. As a result, the channel porosity will be quickly reduced by the growing biofilm volume resulting in a more than proportional pressure drop increase inside the filtration channel, even if the initial pressure drop is lower. This behavior can be seen in the present work for commercial spacer, which has a lower initial pressure drop than the pillar spacer. However, due to non-woven design, the commercial spacer has a tendency to produce higher shear stress on the membrane surface and consequently more biofouling. During the filtration process, the biofilm grows faster for commercial spacer compared to pillar spacer resulting in a lower permeate flux production. Thus for biologically active feed, the initial pressure drop across a filtration channel is not an accurate parameter that can be relied on to predict the filtration performance and energy requirements.

The present work clearly establishes that the design of feed spacer for biologically active feed affects substantially the biofouling development potential, which is ultimately dependent on the shear stress distribution on membrane surface. A low and evenly distributed shear stress is desirable and the presence of local hot-spots in the shear stress profile should essentially be avoided in the engineering of spacer design. An associated minimal channel pressure drop might be an additional advantage for potentially minimizing the overall energy requirements. Moreover, higher operating pressure conditions are also found to be detrimental to filtration performance due to the rapid formation of biofilm in the filtration system. CFD calculations are focused herein on the fluid velocity and shear stress distributions inside the hole-pillar spacer-filled filtration channel. Simultaneously, the biofilm growth is characterized by OCT and correlated to the hydrodynamics state within the channel. In future research works, fluid mass transfer simulations will be targeted to investigate its evolvement with the biofilm growth behavior on UF membrane surface.

## Conclusions

Three spacer designs, including a non-woven commercial spacer, a symmetric pillar, and a hole-pillar spacer are designed and fabricated using a 3D-printer. These spacers are first numerically investigated and later tested on a benchtop UF setup. The outcomes of this study are summarized as follows:

1. The hole-pillar spacer is found to have the lowest initial channel pressure drop, followed by the commercial spacer and pillar spacer, suggesting that hole-pillar spacer has the least initial energy consumption.
2. Local feed velocity is asymmetric for the commercial spacer, while pillar and hole-spacer produce symmetric velocity profiles that have roughly the same average velocity magnitudes. Behind the filament intersection, hole-pillar spacer produces high fluid velocity due to micro-jet formation, which eliminates the dead zones in each spacer cell.
3. Shear stress is highest for the commercial spacer, and lowest for the hole-pillar spacer. Consequently, the highest biofouling is observed for the commercial spacer and lowest for hole-pillar spacer at all applied pressures.
4. Independent of applied pressure, the hole-pillar spacer produces the highest permeate flux. At P = 0.5 bar, the flux gain is 75% compared to the commercial spacer, while at higher applied pressure (P = 1.0 bar), this gain was reduced to 63%.
5. Increasing applied pressure results in larger biofouling compared to lower applied pressure, as revealed by OCT scans.

## Supplementary Information

Supplementary Information

## Data Availability

The datasets generated during and/or analyzed during the current study are available from the corresponding author on reasonable request.
