# Supplementary material for: Novel hole-pillar spacer design for improved hydrodynamics and biofouling mitigation in membrane filtration
Source: Sci Rep. 2021 Mar 26;11:6979. doi: 10.1038/s41598-021-86459-w (PMC7998016; doi:10.1038/s41598-021-86459-w)
Supplement: Supplementary file 1 — Supplementary Information [file 41598_2021_86459_MOESM1_ESM.docx]

Supplementary material

Novel hole-pillar spacer design for improved hydrodynamics and biofouling mitigation in membrane filtration

Adnan Qamar^₶a^, Sarah Kerdi^₶a^, Syed Muztuza Ali^b^, Ho Kyong Shon^b^, Johannes S. Vrouwenvelder^a^, Noreddine Ghaffour^a*^

*^a^ King Abdullah University of Science and Technology (KAUST), Water Desalination and Reuse Center (WDRC), Biological and Environmental Science and Engineering (BESE), Thuwal 23955-6900, Saudi Arabia, Email:*

*^b^ School of Civil and Environmental Engineering, University of Technology, Sydney, Post Box 129, Broadway, NSW 2007, Australia*

*^₶^ Equal Contribution*

*Corresponding author: Noreddine Ghaffour

Email: [*noreddine.ghaffour@kaust.edu.sa*](mailto:noreddine.ghaffour@kaust.edu.sa)

**Computation domain and mesh independence**

For numerical calculations, the computational fluid domains were extracted by subtracting the respective CAD designs (same CAD that were used for 3D-printing) of each spacer with the hollow rectangular channel of 22 mm × 5.1 mm × 1.5 mm (L × W × H) in *SolidWorks* software (Version 2018). The extracted computational fluid volume along with numerical boundary conditions utilized in the simulations is depicted in Fig. S1. At the inlet, the flow velocity is specified and set equal to the average channel velocity of U_o_ = 0.185 m/s (U_o_ = Q/A, where Q is the volumetric feed flow rate and A is the channel inlet cross-section area 15 mm x 1.2 mm) corresponding to the feed flow rate used in the experiments (Q = 200 mL/min). Periodic boundary conditions are used in the spanwise direction of the channel. The typical value of permeate flow ranges is between 1 - 10 μm/s^1^, which is very low compared to the feed cross-flow (0.05% per spacer unit). Thus, a reasonable assumption of wall impermeability can be imposed, allowing to model top and bottom channel walls with no-slip boundary conditions (V = 0). The outflow is kept at a reasonable distance to allow convective disturbances to smooth and avoid spurious backflow pressure generation at the exit. The flow inside the channel is initialized with zero velocity and atmospheric pressure conditions.

The computational domain was discretized into millions of control volumes using a polyhedral surface mesh which transforms to hexahedral mesh away from the solid boundary (ANSYS Mosaic^2^). Polyhedral control volume meshes are known to have a faster and robust converge with a small number of iterations resulting in a rapid solution with greater scalability compared to other different types of meshes^3^. Prior to the simulations, a mesh independence test was performed to gauge the mesh requirement and to ensure that the discretization errors do not influence the accuracy of the solver. As velocity gradients at the walls are hard to resolve, shear stress and flow velocity at two different probe locations are monitored. The shear stress probe is located at the bottom wall and in the center of the channel, while the flow velocity probe is exactly located at the centroid of the channel. The simulations are executed for different grids and these two quantities were measured as presented in Table S1. It can be seen that as the number of mesh points increases, the shear stress and flow velocity reduce (converge) and the difference in the results is found to be less than 0.3 % for any mesh greater than 25 million grid points. Thus, for load balancing and efficient computation, a 26 million mesh was found to be sufficient to resolve the fluid flow accurately.

**Table S1**. Mesh independence study by monitoring wall shear and flow velocity.

| Total mesh | Minimum mesh size  (μm) | Wall shear  (N/mm^2^) | Velocity  (m/s) |
| --- | --- | --- | --- |
| 20 million | 80 | 4.850 | 0.0412 |
| 25 million | 50 | 4.215 | 0.0380 |
| 30 million | 25 | 4.199 | 0.0378 |
| 35 million | 10 | 4.197 | 0.0377 |


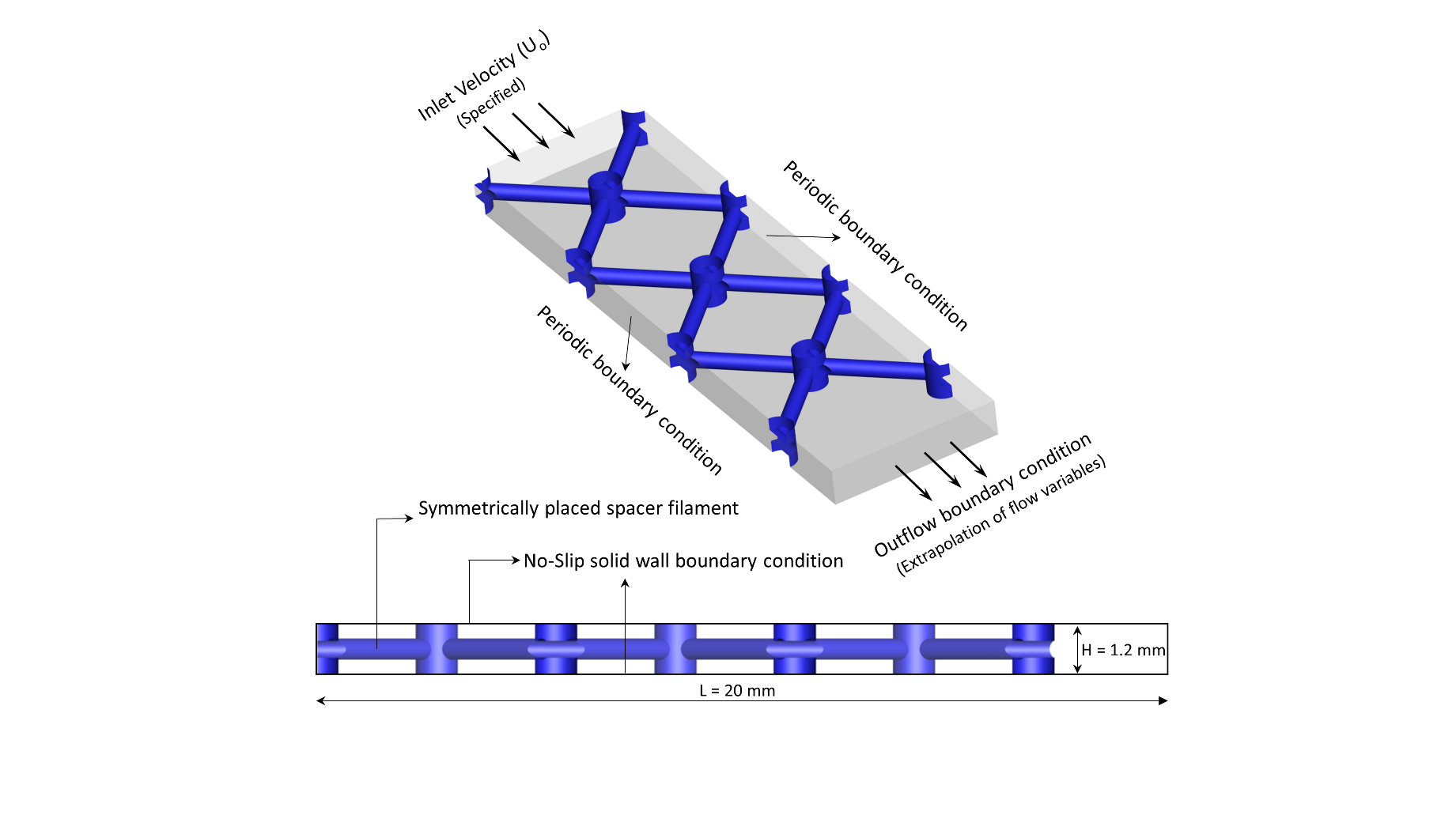


**Figure S1**. Computational domain extracted from the computer aid design (CAD) along with used boundary conditions in the simulation.

**Biofilm characterization by OCT**


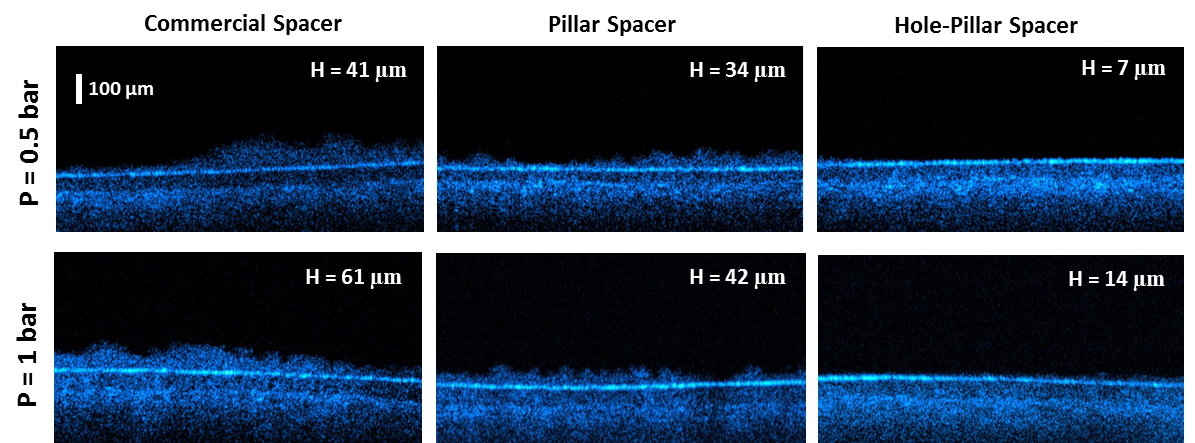


**Figure S2.** OCT scans taken on the membrane surface at a location close to the spacer filament. Biofilm variation on membrane surface for the three tested feed spacers at applied pressures of P = 0.5 bar, and P = 1.0 bar taken at 67 h of filtration process. The scale bars of all images are the same.

**References**

1 Qamar, A., Bucs, S., Picioreanu, C., Vrouwenvelder, J. & Ghaffour, N. Hydrodynamic flow transition dynamics in a spacer filled filtration channel using direct numerical simulation. *J. Membr. Sci.* **590**, 117264 (2019).

2 ANSYS Fluent 19.2, *Meshing User's Guide* (ANSYS, Inc. Southpointe, Canonburg, PA, 2019).

3 ANSYS Release 19.0, *ANSYS Fluent-Theory Guide* (ANSYS, Inc. Southpointe, Canonburg, PA, 2019).
